# Serosurvey of Smooth *Brucella*, *Leptospira* spp. and *Toxoplasma gondii* in Free-Ranging Jaguars (*Panthera onca*) and Domestic Animals from Brazil

**DOI:** 10.1371/journal.pone.0143816

**Published:** 2015-11-25

**Authors:** Mariana Malzoni Furtado, Solange Maria Gennari, Cassia Yumi Ikuta, Anah Tereza de Almeida Jácomo, Zenaide Maria de Morais, Hilda Fátima de Jesus Pena, Grasiela Edith de Oliveira Porfírio, Leandro Silveira, Rahel Sollmann, Gisele Oliveira de Souza, Natália Mundim Tôrres, José Soares Ferreira Neto

**Affiliations:** 1 Jaguar Conservation Fund/ Instituto Onça-Pintada, Mineiros, Goiás, Brasil; 2 Departamento de Medicina Veterinária Preventiva e Saúde Animal, Faculdade de Medicina Veterinária e Zootecnia, Universidade de São Paulo, São Paulo, São Paulo, Brasil; 3 Universidade Católica Dom Bosco, Programa de Pós-Graduação em Ciências Ambientais e Sustentabilidade Agropecuária, Campo Grande, Mato Grosso do Sul, Brasil; 4 Instituto de Biologia, Instituto de Ciências Biomédicas, Universidade Federal de Uberlândia, Uberlândia, Minas Gerais, Brasil; Federal University of Pelotas, BRAZIL

## Abstract

This study investigated the exposure of jaguar populations and domestic animals to smooth *Brucella*, *Leptospira* spp. and *Toxoplasma gondii* in the Cerrado, Pantanal and Amazon biomes of Brazil. Between February 2000 and January 2010, serum samples from 31 jaguars (*Panthera onca*), 1,245 cattle (*Bos taurus*), 168 domestic dogs (*Canis lupus familiaris*) and 29 domestic cats (*Felis catus*) were collected and analysed by rose bengal test for smooth *Brucella*, microscopic agglutination test for *Leptospira* spp. and modified agglutination test for *T*. *gondii*. Cattle populations from all sites (9.88%) were exposed to smooth *Brucella*, but only one jaguar from Cerrado was exposed to this agent. Jaguars captured in the Cerrado (60.0%) and in the Pantanal (45.5%) were seropositive for different serovars of *Leptospira* spp., cattle (72.18%) and domestic dogs (13.1%) from the three sites and one domestic cat from Pantanal were also seropositive for the agent. The most prevalent serotype of *Leptospira* spp. identified in jaguars from the Cerrado (Grippotyphosa) and the Pantanal (Pomona) biomes were distinct from those found in the domestic animals sampled. Jaguars (100%), domestic dogs (38.28%) and domestic cats (82.76%) from the three areas were exposed to *T*. *gondii*. Our results show that brucellosis and leptospirosis could have been transmitted to jaguars by domestic animals; and jaguars probably play an important role in the maintenance of *T*. *gondii* in nature.

## Introduction

Although the impact of pathogens on the population dynamics of wild carnivores remains unknown, the impact of infection and disease on their populations is predicted to grow over the next 50–100 years [1,2]. In the past, infectious diseases have been viewed as natural processes in wild populations [3]. However, fragmentation of natural habitats, increased human settlement around natural areas and the consequent increased contact between domestic and wild animals can increase the occurrence of diseases [3,4]. The transmission of pathogens can occur in both directions: from domestic to wild or wild to domestic animals [5].

Brucella are Gram-negative bacteria usually associated with large losses in cattle herds [6]. In Brazil, it is considered endemic throughout the country [7]. There are few reported incidents in carnivores, and the infection occurs through ingestion of aborted fetuses of infected animals [6]. Cats normally are resistant to *Brucella* spp. and had no clinical disease [6]. But, exposure to *B*. *abortus* has already been described for large cats in captivity, like lions (*Panthera leo*) in the United States [8] and jaguars (*Panthera onca*) in Chile [9] and Brazil [10].

Leptospira are Gram-negative bacteria documented on all continents, mainly in tropical and subtropical regions and in seasons of high rainfall levels [11,12]. Small wild mammals are the main reservoirs of leptospirosis in nature [13,14], but cattle (*Bos taurus*), pigs (*Sus domesticus*) and dogs (*Canis lupus familiaris*) are also considered reservoirs of the agent [11,12]. In Brazil, there are reports of exposure to *Leptospira* spp. in free-ranging felids like ocelots (*Leopardus pardalis*), pumas (*Puma concolor*) and jaguars [15,16].

*Toxoplasma gondii* is an obligate intracellular protozoan able to infect virtually all warm-blooded species worldwide [17,18]. Members of the Family Felidae, both domestic and wild, are essential to the life cycle of *T*. *gondii*, being the only definitive hosts of the parasite [17]. Felids of the *Panthera* genus have a high frequency of exposure to the agent, as described in lions, tigers (*Panthera tigris*), leopards (*Panthera pardus*) and jaguars [16,19–24].

The jaguar (*Panthera onca)* is the largest feline in the Americas and is globally classified as Near Threatened [25]. It is a top predator that plays an important role in ecosystem balance, regulating its prey populations [26]. In order to increase the knowledge of circulation of smooth *Brucella*, *Leptospira* spp. and *T*. *gondii* and verify possible transmission between wild and domestic animals this study used serology to detected antibody against these pathogens in free-ranging jaguars, cattle, domestic dogs and domestic cats (*Felis catus*) from Brazilian Cerrado, Pantanal and Amazon.

## Material and Methods

### Study area

This study was conducted in Brazil, in three different biomes: Emas National Park (ENP) (-18,061146 S; – 52,941067 W) is one of the largest preserved areas of the central Brazilian Cerrado savanna. The region surrounding the park consists of extensive crop plantations and, to a lesser extent, livestock pasture. The Caiman Ecological Refuge (-19, 80319 S; -56,27373 W) and Barranco Alto Ranch (-19,57643 S; – 56,16144 W) are located in the Pantanal, the largest wetland in the world. Rural properties there engage predominantly in extensive cattle ranching and in some cases, ecotourism. Cantao State Park (CSP) (-9,64503 S; -50,13065 W) is situated in the transitional area between the Cerrado and Amazon biomes.The main economic activity on surrounding rural properties is extensive livestock ranching. Also, indigenous lands are found near this park.

### Biological samples

Between February 2000 and May 2009, 30 free-ranging jaguars were captured across the three study areas (5 in ENP, 22 in the Pantanal and 3 in CSP). We used two techniques to capture jaguars: trained hounds and metal cage traps [27]. In addition, one juvenile jaguar raised by humans on indigenous lands near CSP was included in this survey.

The jaguars were anesthetized intramuscularly with a combination of tiletamine-zolazepam (Zoletil^®^ or Telazol^®^), with an average dose of 9.7 mg/kg. Blood samples were taken by internal femoral vein puncture in vacuum tubes without anticoagulant, and physical examinations were conducted. Thirteen individuals were recaptured at intervals of 60 days or more. All adult jaguars were fitted with radiocollars and monitored using radiotelemetry or camera traps. The monitoring period comprised the interval between its capture day and the last location obtained until November 2008, except one animal that was monitored until February 2010.

Between May 2008 and January 2010, blood samples were collected from 1245 cattle (465 in ENP, 356 in the Pantanal and 424 in CSP), 168 domestic dogs (83 in ENP, 29 in the Pantanal and 56 in CSP) and 29 domestic cats (9 in ENP, 10 in the Pantanal and 10 in CSP) from rural properties bordering the preserved areas sampled. Cattle sampled were mostly females aged ≥ 24 months (n = 1188), only on one farm surrounding the ENP we sampled males (n = 57). Blood samples of cattle were collected from the tail vein and blood samples of dogs and cats were collected by jugular or cephalic vein puncture in vacuum tubes without anticoagulant.

The samples were transported to the field laboratory, where blood was centrifuged for 5 minutes at 1,200 *g*. The serum was removed from the clot tube, separated in aliquots and stored at -20°C.

Handling procedures agreed with Ethical Principles in Animal Research adopted by the Bioethic Commission of the School of Veterinary Medicine and Animal Science of University of São Paulo (FMVZ-USP) and was approved by the permit number 1471/2008. Instituto Chico Mendes de Conservação da Biodiversidade (ICMBio) granted field permits to work in ENP, Pantanal and CSP (Permits number 14637, 11214 and 11628, respectively).

### Laboratory analyses

We surveyed the exposure of jaguars, cattle and dogs to smooth *Brucella*; and jaguars, cattle, dogs and cats for *Leptospira* spp. and *T*. *gondii*. Due to limited serum quantities, not all tests were performed on all individuals.

Antibodies against smooth *Brucella* were detected using the rose bengal test and *Brucella abortus* (stain 1119–3) as antigen [7]. Serum from cattle previously known to be infected with *B*. *abortus* was used as positive control. Even though cats were considered resistant to Brucella spp., jaguars of the three study areas feed on animals that could be infected (like cattle, white-lipped peccary, and collared peccary).

Sera were tested for antibodies against *Leptospira* spp. using the microscopic agglutination test [28] against the serovars Andamana, Australis, Autummnalis, Bataviae, Bratislava, Butembo, Canicola, Castellonis, Copenhageni, Cynopteri, Grippotyphosa, Hardjo, Hebdomadis, Icterohaemorrhagiae, Javanica, Panama, Patoc, Pomona, Pyrogenes, Sentot, Shermani, Tarassovi, Whitcombi and Wolffi. The sera used as positive controls were produced in the Department of Preventive Veterinary Medicine and Animal Health of FMVZ-USP. We considered any animal reagent for one or more serovars as seropositive. To determine the most prevalent serovar, we considered the serovar with the highest titer. Individuals who had two or more serovars with identical titers were excluded from this analysis. However, these animals were considered seropositive for at least one serovar in frequency of positivity [29]. The most prevalent serovar were calculated by species and study area.

For detection of *T*. *gondii* antibodies in jaguars we used the Modified Agglutination Test (MAT) [30]. The MAT was chosen for jaguars, since it does not require a specific conjugate [31]. Individuals with titers equal or greater than 25 were considered positive. For domestic animals we used the indirect fluorescent antibody test (IFAT) [32]. For cattle, we used 64 as cut off value and for dogs and cats, 16. The positive samples were further diluted to obtain the maximum titer of the reaction. Sera from cattle, dog and cat previously known to be infected with *T*. *gondii* were used as positive controls.

### Data processing and statistical analysis

The diagnoses were presented according to species and study area sampled. For statistical analysis, the diagnoses of recaptured jaguars were interpreted in parallel (an animal that had at least one positive result was considered positive), avoiding pseudorreplication and ensuring data independence [33].

The jaguar monitoring data allowed the characterization of the spatial distribution of pathogens in the study areas. The property with at least one domestic animal diagnosed positive for a particular pathogen was considered a focus of infection.

We used a logistic regression to compare the diagnoses obtained for different species. The models were implemented using the software R, version 2.10.1 [34]. To compare diagnoses of different study areas, jaguars and domestic animals were grouped. A table with the coefficient estimates, their standard errors (SE), 95% confidence intervals (CI95) and *p* values for each model applied is presented. Coefficients with *p* values < 0.05 were considered having a significant effect on the probability of an individual to test positive.

## Results

Most of the 31 sampled jaguars (96.8%) showed excellent or good physical conditions, appropriate body weight and absence of any clinical signs of diseases.

**Fig 1:**
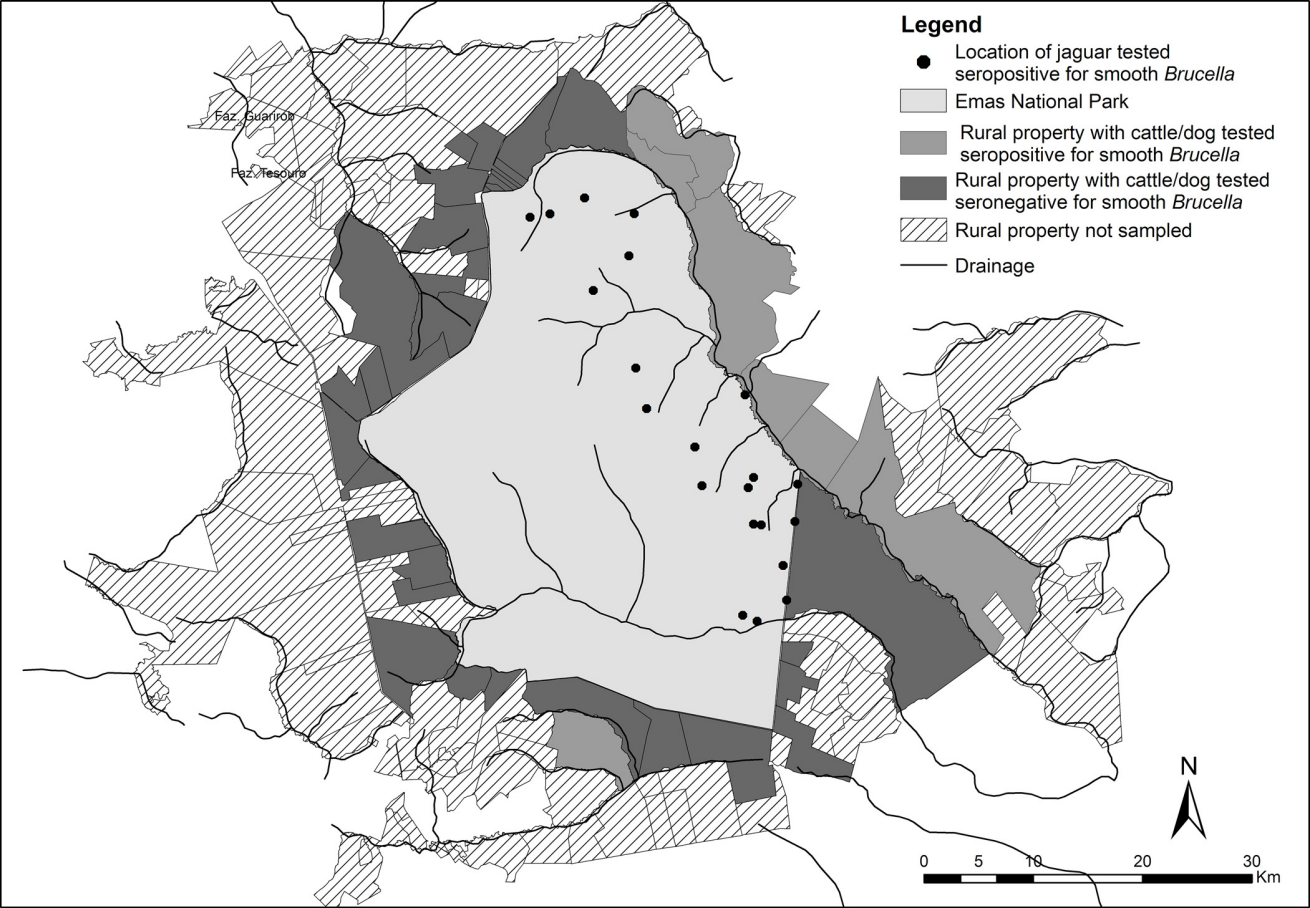
Locations obtained through radiotelemetry and camera trap of jaguar tested seropositive for smooth *Brucella* and rural properties with cattle/dog tested seropositive for smooth Brucela in the region of Emas National Park.

Results for smooth *Brucella* are organized in Table 1. A single jaguar from ENP was seropositive for smooth *Brucella*. This animal was captured twice and only in the recapture event, approximately 60 days after its initial capture, was it found to be seropositive. All rural properties in the Pantanal and CSP, where cattle were sampled, had at least one seropositive individual for smooth *Brucella*, and dogs from one rural property in the Pantanal were seropositive for this agent. According to statistical analysis, individuals sampled from ENP region were more exposed to smooth *Brucella* than individuals from CSP region (*p* = 0.032), and cattle were significantly more exposed to the agent than domestic dogs (*p* = 0.014). No other significant associations were detected (Table 2). The movement of the jaguar captured in ENP that tested seropositive for smooth *Brucella* is shown in Fig 1.

**Table 1:** Results for smooth *Brucella* by study area and species samples between February 2000 and January 2010.

		Emas National Park			Pantanal			Cantão State Park		
Species	Category	Examined	Positive	%	Examined	Positive	%	Examined	Positive	%
**Jaguar**	animals	5	1	20.0	22	0	0	4	0	0
**Cattle**	Properties	9	6	66.7	7	7	100.0	6	6	100.0
	animals	465	53	11.4	356	39	11.0	424	31	7.3
**Domestic dog**	Properties	25	0	0	6	1	16.7	13	0	0
	animals	48	0	0	22	2	9.1	39	0	0

**Table 2:** Parameters estimated by logistic regression testing the effect of the species sampled (coeficient β_1_) and study site (coeficient β_2_) for the diagnosis of smooth *Brucellas*. Using as reference category for species: cattle, and for study site: Emas National Park.

Parameters	Estimated	Standard Error	*P value*	CI 95%
Intercept	-2.055	0.145	<0.001	[-2.351; -1.780]
β_1_ (domestic dog)	-1.767	0.721	0.014	[-3.577; -0.600]
β_1_ (jaguar)	-1.280	1.025	0.212	[-4.168; 0.286]
β_2_ (Pantanal)	-0.018	0.220	0.936	[-0.454; 0.412]
β_2_ (CSP)	-0.506	0.236	0.032	[-0.977; -0.050]

**Fig 2:**
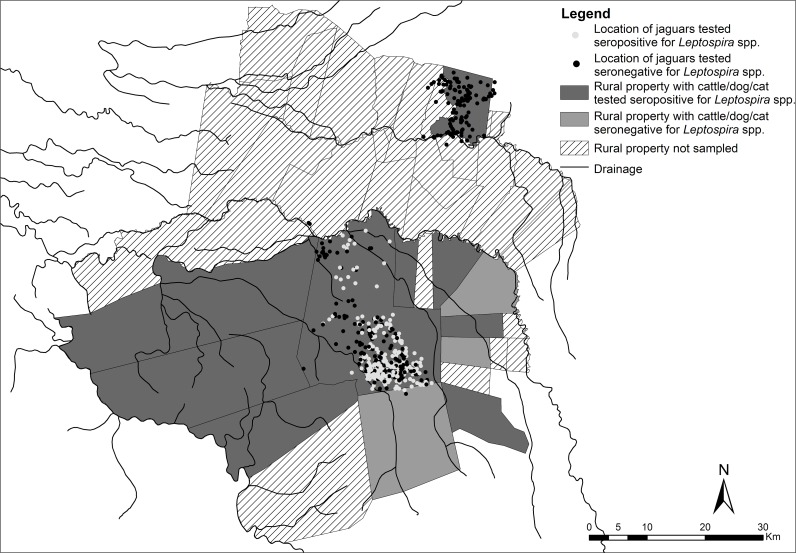
Locations obtained through radiotelemetry of jaguars tested seropositive and seronegative for *Leptospira* spp. and rural properties with cattle/dog/cat tested seropositive for *Leptospira* spp. in the region of Pantanal.

Results for *Leptospira* spp. are organized in Table 3, with individuals seropositive for any serovar of *Leptospira* spp. and the most prevalent serotype diagnosed for each species. Jaguars captured in ENP and in the Pantanal were seropositive for different serovars of *Leptospira* spp. with titers ranging between 100 and 6400, while the four jaguars from CSP were negative for the agent. The most prevalent serovars found in jaguars were Grippothyphosa in ENP and Pomona in the Pantanal. Cattle presented frequencies of 68.4% (Pantanal) to 73.7% (ENP) of positivity for *Leptospira* spp., with the serovar Hardjo being the most infective serovar for the species in all three sites. The seropositivity of dogs ranged from 7.2% in ENP to 24.1% in the Pantanal. Only one cat sampled in the Pantanal was seropositive. Cattle were significantly more exposed to *Leptospira* spp. than domestic dogs (*p*<0.001), cats (*p*<0.001) and jaguars (*p*<0.001). No other significant associations were detected (Table 4). Fig 2 illustrates the properties that were focus of leptospirosis and movement of jaguars in the Pantanal.

**Table 3:** Results of serologic tests for *Leptospira* spp. and most prevalent serovar, by study areas and species sampled between February 2000 and January 2010.

		Emas National Park				Pantanal				Cantão State Park			
Species	Category	Exam.^a^	Positive	%	Serovar^b^	Exam.	Positive	%	Serovar	Exam.	Positive	%	Serovar
**Jaguar**	animals	5	3	60.0	Grippotyphosa	22	10	45.5	Pomona	4	0	0	
**Cattle**	Properties	9	9	100.0	Hardjo	7	7	100.0	Hardjo	6	6	100.0	Hardjo
	Animals	464	342	73.7		345	236	68.4		424	312	73.6	
**Domestic dog**	Properties	34	5	14.7	Autumnalis	7	3	42.9	Canicola	16	6	37.5	Hardjo
	Animals	83	6	7.2		29	7	24.1		56	9	16.1	
**Domestic cat**	Properties	8	0	0	-	6	1	16.7	Hardjo	6	0	0	-
	Animals	9	0	0		10	1	10.0		10	0	0	

^a^Examined

^b^Most prevalent serovar

**Table 4:** Parameters estimated by logistic regression testing the effect of the sampled species (coeficient β_1_) and study site (coeficient β_2_) for the diagnosis of *Leptospira* spp. Using as reference category for species: cattle, and for study site: Emas National Park.

Parameters	Estimated	Standard Error	*P value*	CI 95%
Intercept	0.978	0.101	<0.001	[0.782; 1.179]
β_1_ (domestic dog)	-2.862	0.238	<0.001	[-3.354; -2.416]
β_1_ (domestic cat)	-4.281	1.019	<0.001	[-7.164; -2.731]
β_1_ (jaguar)	-1.273	0.369	<0.001	[-2.019; -0.557]
β_2_ (Pantanal)	-0.129	0.150	0.388	[-0.423; 0.165]
β_2_ (CSP)	0.038	0.144	0.793	[-0.244; 0.320]

Results for *T*. *gondii* are organized in Table 5, presenting the variation of antibody titers found. All jaguars of the three study sites were seropositive for *T*. *gondii* with antibody titers between 25 and 3200. Cattle presented a seropositivity below 1.0% in the three areas, dogs presented seropositivity between 29.1 and 47.8%, and cats between 77.8 and 90.0%. Cats and dogs were significantly more exposed to *T*. *gondii* than cattle (*p*<0.001). No other significantly associations were detected (Table 6). Jaguars were not included in the analysis due to its 100% exposure to *T*. *gondii*.

**Table 5:** Results for *Toxoplasma gondii* and variation of antibodies titer found, by study areas and species sampled between February 2000 and January 2010. Using as cut off for jaguars, titer = 25, for cattle, 64 and for dogs and cats, 16.

		Emas National Park				Pantanal				Cantão State Park			
Species	Category	Exam.^a^	Pos.^b^	%	Titers^c^	Exam.	Pos.	%	Titers	Exam.	Pos.	%	Titers
**Jaguar**	Animals	5	5	100.0	200	22	22	100.0	25–3200	4	4	100.0	200–3200
**Cattle**	Properties	9	2	22.2	64–128	7	2	28.6	128	6	1	16.7	256
	Animals	454	2	0.4		348	2	0.6		422	1	0.2	
**Domestic dog**	Properties	26	13	50.0	16–512	7	7	100.0	16–2048	14	11	78.6	16–512
	Animals	55	16	29.1		27	11	40.7		46	22	47.8	
**Domestic cat**	Properties	8	6	75.0	16–1024	6	5	83.3	32–2048	6	5	83.3	32–2048
	Animals	9	7	77.8		10	9	90.0		10	8	80.0	

^a^Examined.

^b^Positive.

^c^Variation of antibodies titer

**Table 6:** Parameters estimated by logistic regression testing the effect of the sampled species (coeficient β_1_) and study site (coeficient β_2_) for the diagnosis of *Toxoplasma gondii*. Using as reference category for species: cattle, and for study site: Emas National Park.^a^

Parameters	Estimative	Standard Error	*P value*	CI 95%
Intercept	-5.895	0.514	<0.001	[-7.028; -4.985]
β_1_ (domestic dog)	5.078	0.487	<0.001	[4.214; 6.162]
β_1_ (domestic cat)	7.091	0.669	<0.001	[5.879; 8.531]
β_2_ (Pantanal)	0.564	0.419	0.179	[-0.260; 1.390]
β_2_ (CSP)	0.587	0.371	0.114	[-0.137; 1.324]

^a^Model not identified for jaguars.

## Discussion

### Smooth *Brucella*

We chose the AAT test to be the most suitable for diagnosis of smooth *Brucella* in wild animals [35]. Although the cattle populations in the three areas of study showed exposure to smooth *Brucella*, only one jaguar captured in ENP was seropositive. The positivity of smooth *Brucella* may be due to infection by *B*. *abortus*, *B*. *suis* or *B*. *mellitensis* [6], the latter being considered exotic in Brazil [36].

This is the first report of exposure of a free-ranging jaguar in the Cerrado biome to smooth *Brucella*. Jaguars seropositive for brucellosis by the AAT test have already been reported in free-ranging animals in the Brazilian Atlantic Forest [37] and in captivity in Chile and Brazil [9,10]. Almeida et al. [10] detected DNA of *B*. *abortus* and *B*. *canis* in captive jaguars from Brazil. The jaguar exposed to the agent in this study used the areas near the border of ENP, with farms that practice extensive livestock ranching, as part of its home range (Fig 1). The surroundings of the three study areas consist of farms with significant frequency of cattle that is seropositive for brucellosis, favoring transmission of the disease to wild predators. Interestingly, cattle predation by jaguars surrounding ENP was reported less frequently than in the other two areas.

The monitoring of the jaguar seropositive for brucellosis for 12 months showed no change in movement patterns (Fig 1), and based on camera trap pictures it remained in excellent physical condition with no apparent manifestations of disease.

Bovine brucelosis caused by *B*. *abortus* is the most prevalent infection caused by *Brucella* in Brazil, and the positive results in cattle can unequivocally be attributed to *B*. *abortus* [7]. The cattle of the three study areas showed significant rates of *B*. *abortus* infections. Domestic dogs were exposed to smooth *Brucella* but the role of dogs in the transmission of this agent is negligible [38].

Regarding the statistical results, the greater exposure of cattle to *B*. *abortus* than dogs was expected, since cattle are considered the natural hosts of the agent [39]. However, the absence of a significant difference in exposure to smooth *Brucella* betwen cattle and jaguars is probably related to the small number of jaguars sampled. Therefore, these results should be interpreted with caution.

These results show that brucellosis is endemic in cattle populations in the surroundings of preserved areas studied. Jaguars, like other carnivores, have low risk of infection to the agent and probably have no important role in the maintenance of smooth *Brucella* in the study sites.

### *Leptospira* spp.

Jaguars from ENP and the Pantanal were exposed to *Leptospira* spp., and so were cattle and dogs of the three study areas, and a domestic cat from the Pantanal. The absence of seropositive jaguars for *Leptospira* spp. in the CSP was unexpected, because the pathogen is transmitted and survives well in humid environments such as those found in the CSP. Also, the high levels of infection in cattle in this region indicated that the environment is conducive to the transmission of leptospirosis.

Unlike the jaguars from CSP, the jaguars from ENP and the Pantanal showed high exposure to *Leptospira* spp. The most prevalent serovar found in the Pantanal was the same diagnosed by Nava [37] in free-ranging jaguars in the Atlantic Forest (serovar Pomona). The serovar Hardjo was previously reported in jaguars kept in captivity [40,41] and the sevorar Canicola in free-ranging jaguars [42], both from Brazil.

The transmission of leptospirosis can occur through water and soil contaminated by urine of an actively infected animal [11], or by eating infected animals [43]. In ENP, the seropositive jaguars moved mainly along watercourses (Fig 2), which may have favored the indirect transmission and high exposure to the agent. The Pomona serovar, which was the most prevalent serovar in many jaguars from the Pantanal, has cattle and domestic pigs as its main hosts [11], but was found at low frequency in cattle examined in this study. While the properties in the Pantanal had domestic pigs, these animals were not sampled.

Some jaguars had high titers of antibodies against *Leptospira* spp., indicating recent or active infections. However, monitoring of these animals by radiotelemetry showed no changes in their movement patterns, suggesting that jaguars probably do not show clinical signs for leptospirosis, as proposed Lilenbaum et al. [44]. Corrêa et al. [45] suggest that the Felidae Family is more resistant to leptospirosis because rodents constitute an important source of prey, making it resistant to disease caused by the pathogen.

The presence of at least one bovine seropositive for *Leptospira* spp. in each of the sampled properties indicated the widespread dissemination of the agent in the three areas. The frequency of seropositive dogs, apparently higher in the Pantanal and CSP than ENP, is probably related to climate characteristics of the CSP and Pantanal, which have high humidity, and pattern of seasonal flooding, favoring the epidemiology and spread of the pathogen, which can survive for long periods in water or moist soil [12].

The low frequency or absence of *Leptospira* in cats in this study is in agreement with the observations of Acha and Szyfres [11] and Vijayachari et al. [12] that the agent is rarely found in this species.

The results suggest that the cattle were more exposed to *Leptospira* spp. than the other species examined, they are considered the maintenance host of serovar Hardjo. As the most prevalent serovar found in jaguars were distinct from those detected in cattle, dogs and cats, the epidemiology of leptospirosis in jaguars probably does not involve these domestic animals as reservoirs. However, especially in the Pantanal region, the presence of *Leptospira* spp. should be investigated in domestic pigs.

### *Toxoplasma gondii*

All jaguars from the three preserved areas were exposed to *T*. *gondii*. Cats and dogs had significant exposure to the agent and only five of 1224 cattle examined were classified seropositive.

The seropositivity for *T*. *gondii* in all jaguars and in all capture events suggests that the species is a definitive host of the agent in the wild in the three study areas–agreeing with Demar et al. [46], who made the same affirmation after isolating *T*. *gondii* in a free-ranging jaguar in French Guiana. Exposure to *T*. *gondii* has already been reported in jaguars in Brazil [16,19,24,47,48]. Just like in cats, jaguars seropositive for *T*. *gondii* at some point in their lives release oocysts into the environment and can be constantly reexposed to the agent. However, the number of oocysts released by a wild feline is lower than that released by domestic cats [49].

Most antibody titers found in jaguars were below the titer of 4,000 reported for the free-ranging jaguar from French Guiana [46]. Comparing with jaguars in captivity, the wild animals from this study presented higher titers than those reported by Silva et al. [19], Spencer et al. [20], De Camps et al. [21] and Pimentel et al. [48], which were ≤ 100. The difference is probably related to dietary and behavioral differences between captive and free-ranging animals.

Although little is known about the role of this pathogen in the mortality and morbidity of wild felines [19] no jaguar sampled in this study showed clinical signs of toxoplasmosis. Good general condition of the animals at the time of capture and monitoring of individuals by radiotelemetry confirmed that observation.

The exposure of cattle to *T*. *gondii* in the three areas of study seemed to be lower than exposure rates described for the Brazilian territory by several authors [50–53]. The percentage of seropositive dogs found on rural properties in the Pantanal (40.7%) was similar to that reported by Marques et al. [54] in dogs in rural area of Mato Grosso do Sul (47.61%). In Goiás, where ENP is located, Fernandes and Barbosa [55] observed a frequency of 57.1% of seropositive dogs, and in the present study the frequency of seropositive dogs in the surroundings of ENP was 29.1%. The dogs can act as intermediate and mechanical host of *T*. *gondii*, but alone, they are not able to maintain the agent in nature [56].

The high percentage of domestic cats seropositives for *T*. *gondii* indicate, as expected, that the species probably helps to maintain the current agent in the three areas of study. Wild and domestic cats are the only definitive hosts of *T*. *gondii* able to release oocysts by feces [17]. High frequency of seropositivity in domestic cats has already been reported in Brazil by Carletti et al. [57], Dubey [58] and Cavalcante et al. [59].

There was no statistically significant difference in exposure to *T*. *gondii* in the three study areas, indicating that the pathogen is equally widespread in these regions. The higher prevalence of the agent in dogs and cats compared to cattle suggests that the spread of infection by sporulated oocysts intake is less efficient than the ingestion of tissue cysts.

The results suggest the existence of a sylvatic clycle of toxoplasmosis where jaguars have an important role in the release of oocysts into the environment, allowing the infection and formation of tissue cysts in their prey. Probably, this cycle remains independent of the domestic cycle.
